# Imaging on the painful and compressed nerve: upper extremity

**DOI:** 10.1007/s00264-025-06436-0

**Published:** 2025-02-10

**Authors:** Marcelo Bordalo, Maria Lua Sampaio Gulde, Elisabet Hagert

**Affiliations:** Aspetar Orthopedic and Sports Medicine Hospital, Doha, Qatar

**Keywords:** Compressive neuropathy, Peripheral nerve, MRI, US

## Abstract

Compressive neuropathies of the upper extremity are a common cause of pain, weakness, and functional impairment, often resulting from chronic mechanical compression or entrapment of peripheral nerves in anatomical regions such as osteofibrous tunnels, fibrous bands, or muscular pathways. While traditional diagnostic methods, including clinical evaluation and electrophysiological studies, are essential, they are limited in localizing lesions and identifying underlying causes. Advances in ultrasonography (US) and magnetic resonance imaging (MRI), particularly MR neurography and high-resolution 3D volumetric imaging, have significantly improved the evaluation of peripheral nerves by enabling detailed visualization of nerve anatomy, adjacent structures, and muscle denervation patterns. This article reviews the role of these imaging techniques in diagnosing and managing compressive neuropathies affecting the brachial plexus, suprascapular, axillary, median, ulnar, and radial nerves, highlighting key imaging findings such as nerve thickening, signal abnormalities, and muscle changes. The integration of advanced imaging modalities into clinical practice enhances diagnostic accuracy, facilitates surgical planning, and improves treatment outcomes for patients with peripheral nerve compression.

## Introduction

Compressive neuropathies affecting the upper extremity are a common source of pain, reduced functionality, and muscular weakness. These disorders result from persistent mechanical pressure or entrapment of peripheral nerves in susceptible anatomical areas, such as osteofibrous tunnels, fibrous structures, or between muscle layers. Prolonged nerve compression may cause progressive ischaemia, neural scarring, and muscle denervation if not promptly diagnosed and managed.

Although clinical assessment, patient history, and electrophysiological testing remain integral to diagnosis, these approaches often face challenges in precisely localizing the lesion and identifying its aetiology. Recent advancements in ultrasonography (US) and magnetic resonance imaging (MRI) have greatly enhanced the evaluation of peripheral nerves by providing high-resolution images of neural anatomy, surrounding soft tissues, and adjacent structures. MRI, in particular, plays a crucial role in detecting nerve thickening, signal abnormalities, and evidence of muscle denervation, offering a valuable, noninvasive alternative to electrodiagnostic studies.

This review article examines the importance of imaging in diagnosing painful and compressed nerves in the upper limb. It delves into specific compressive neuropathies, such as those affecting the brachial plexus, suprascapular nerve, axillary nerve, median nerve, ulnar nerve, and radial nerve. The clinical utility of MR neurography and high-resolution US is thoroughly reviewed, alongside key imaging features that contribute to diagnosis, pre-surgical planning, and monitoring treatment effectiveness.

## Compressive neuropathies

There are anatomical regions in which segments of peripheral nerves are vulnerable or predisposed to becoming trapped or suffering chronic compression. Neural compression occurs especially in osteofibrous tunnels, but it can occur at points where the peripheral nerve passes between muscles or through a band of fibrous tissue.

In changes caused by chronic compression, damage to the nerve occurs progressively or intermittently, sometimes with periods of remission and exacerbation. As a result of repeated trauma or chronic ischaemia, fibrosis can occur within and around neural fascicles. In more serious cases, the nerve fibres may be destroyed, and the neural trunk may be replaced by fibrosis if surgical decompression is not performed. The diagnosis of compression neuropathies is traditionally made based on clinical history findings, physical examination, and electrodiagnostic studies, including electromyography, neural conduction studies and somatosensory evoked potentials. US and MRI have frequently been used to complement the investigation of these neural compressions, although they are not always able to directly demonstrate the cause of compression. When using these two imaging methods, knowledge of the basic anatomy of peripheral nerves and innervation territories is essential. The relative advantage of US is that it allows examination of the entire limb or a large part of the limb relatively quickly, as long as a radiologist is trained to evaluate peripheral nerves. More recently, the possibility of imaging tests, particularly via MRI, to identify signs of muscle denervation in the specific territory of a given nerve or branch has been emphasized. MRI has greater sensitivity in identifying muscle changes and has the advantage of being noninvasive compared to electromyography.

## Specific compressive neuropathies

### Brachial plexus

**Fig. 1:**
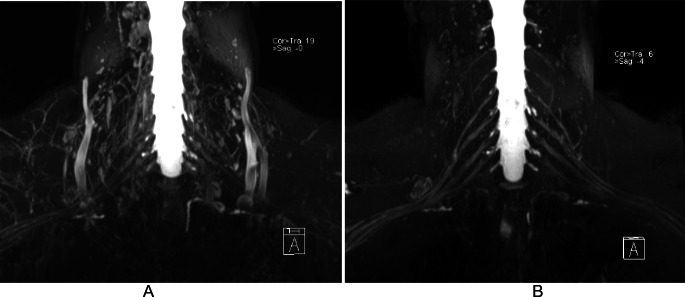
Value of post-contrast on 3D-STIR images of the brachial plexus. MIP reformation of a volumetric SPACE STIR acquisition before (**A**) and after (**B**) intravenous paramagnetic contrast administration. Note the almost complete disappearance of vascular structures surrounding the brachial plexus

Traumatic brachial plexus injuries may occur in high-energy trauma, usually involving motor vehicles, or in neonatal injuries, due to the position of the newborn’s arm during delivery [1]. In adults, traumatic injuries are divided into preganglionic and postganglionic. MR neurography using steady-state acquisition (FIESTA), 3D volumetric images with multiplanar / MIP reconstructions, and DW images has comparable accuracy with CT myelography, with the advantage of allowing postganglionic evaluation by MR. High-contrast volumetric images, based on sampling perfection with application-optimized contrasts using different flip-angle evolutions (SPACE), enables evaluation of the trunks and cords. However, the definition of the brachial plexus may not be clear due to overlap with vessels. The T1 relaxation time of blood is known to be as reduced as that of fat tissue after gadolinium contrast administration in STIR sequences. Consequently, the performance of 3D-STIR sequences with paramagnetic contrast provides a better definition of the brachial plexus outline (Fig. 1) [2–6].

**Fig. 2:**
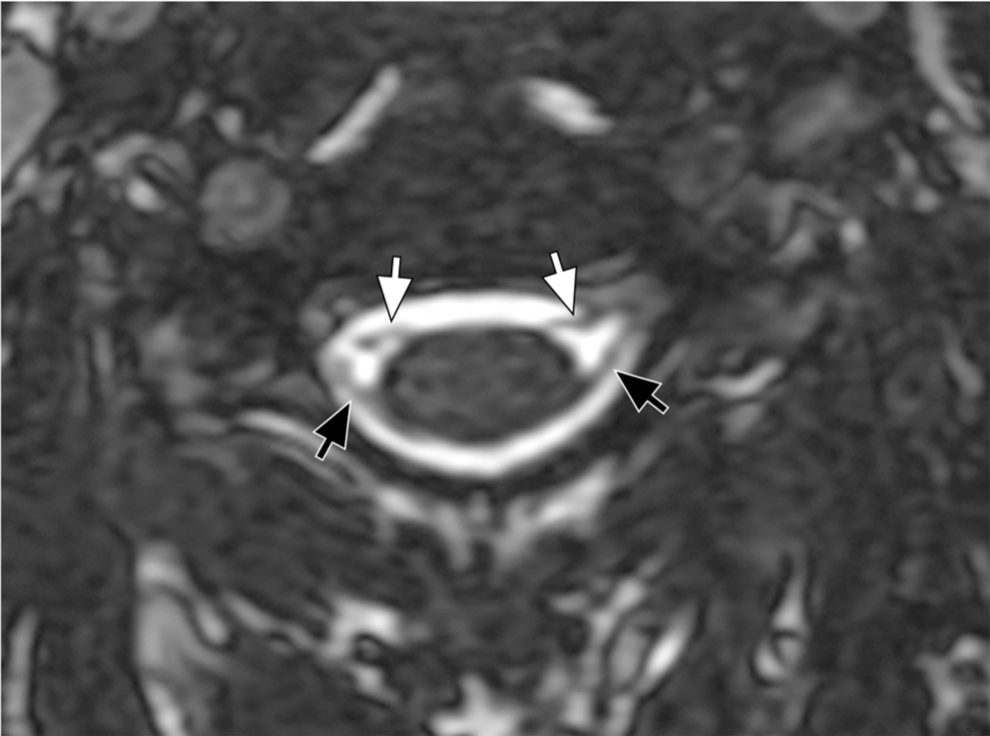
Axial 3D FIESTA image of the cervical spine shows the ventral (white arrows) and dorsal (black arrows) nerve roots emerging from the spinal cord

**Fig. 3:**
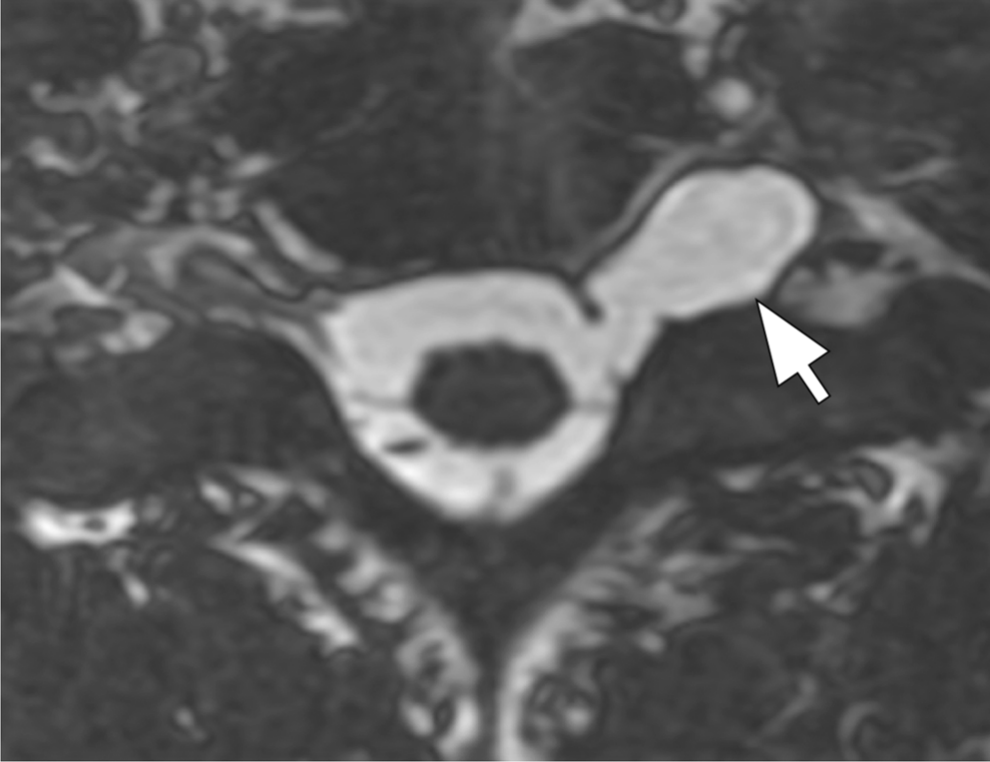
Pseudomeningocele. Axial 3D FIESTA image of the cervical spine demonstrates a pseudomeningocele extending to the left extraforaminal region

**Fig. 4:**
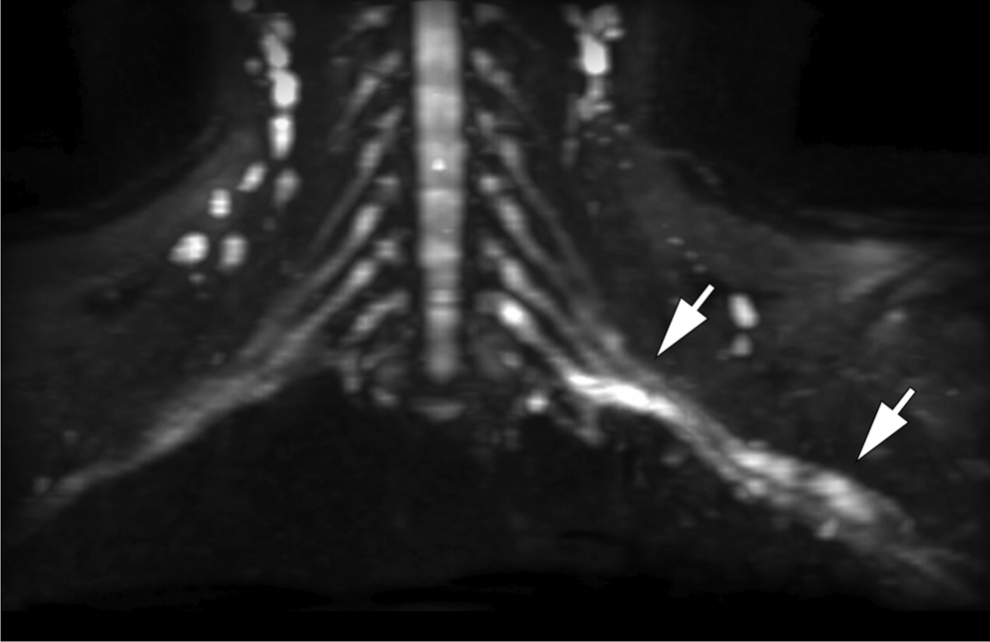
Postganglionar rupture. MIP coronal reformation of a diffusion-weighted acquisition of the brachial plexus (b-value, 150 s/mm^2^). There is thickening and increased signal of the left brachial plexus at the level of the cords (arrows). A Postganglionar rupture was confirmed at surgery

Steady-state sequences are used to diagnose preganglionic avulsions and are comparable to CT myelography (Fig. 2). Pseudomeningoceles are associated with nerve root avulsions in approximately 90% of the cases and are an important red flag for the presence of avulsion (Fig. 3) [7–12]. Postganglionic evaluation is performed on sagittal and coronal fluid-sensitive images. In general, nerve thickening and enhancement are seen in all types of postganglionic lesions (Fig. 4).

**Fig. 5:**
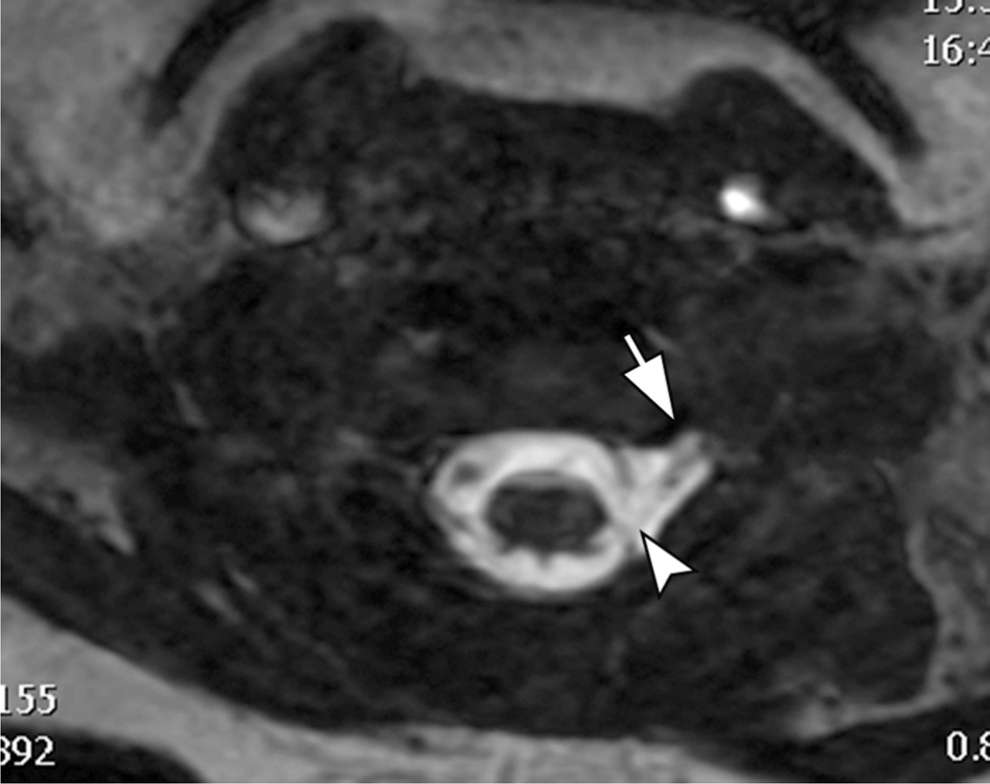
Axial 3D FIESTA image of the cervical spine shows complete preganglionar avulsion of the left ventral and dorsal nerve roots (arrowheads) and a small pseudomeningocele (arrow) extending to the right foramina

Traumatic injuries in neonates are observed in approximately 0.5% of live births. The brachial plexus is most frequently affected, particularly the superior portion involving the C5 and C6 roots, and occasionally C7. However, the inferior portion, including C8 and T1, can also be impacted. MRI plays a valuable role in identifying preganglionic nerve root avulsions. (Fig. 5) [3, 13–16].

**Fig. 6:**
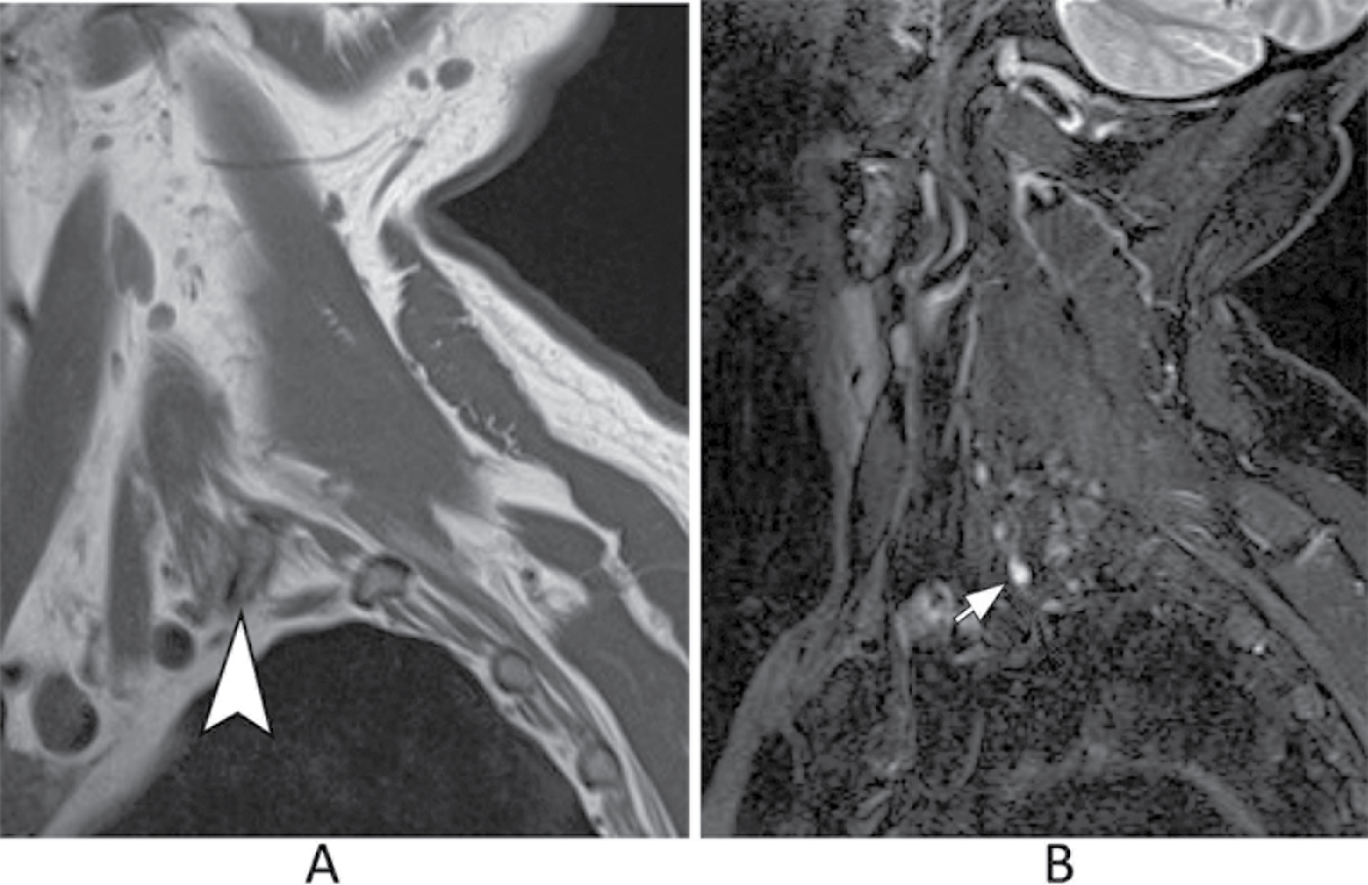
Thoracic outlet syndrome. Sagittal T1-weighted (**A**) and T2-weighted with fat suppression (**B**) images of the brachial plexus. There is a cervical rib (arrowhead) associated with thickening and increased signal of C8 nerve root, indicating neuropathy

Thoracic outlet syndrome (TOS) occurs when the brachial plexus is compressed at one of three potential sites along its pathway: the interscalene triangle, costoclavicular space, or retropectoral space. This condition can also involve vascular structures. The primary causes of compression include congenital fibrous bands associated with the first thoracic rib, the presence of a cervical rib, or muscle hypertrophy. To enhance the assessment of nerve compression and its relationship with adjacent anatomical structures, 3D volumetric imaging combined with postural maneuvers, such as arm elevation, can be utilized. (Fig. 6) [17–22].

### Suprascapular nerve

The most common cause of suprascapular nerve injury is nerve entrapment in the suprascapular and / or spinoglenoid notch [23].

**Fig. 7:**
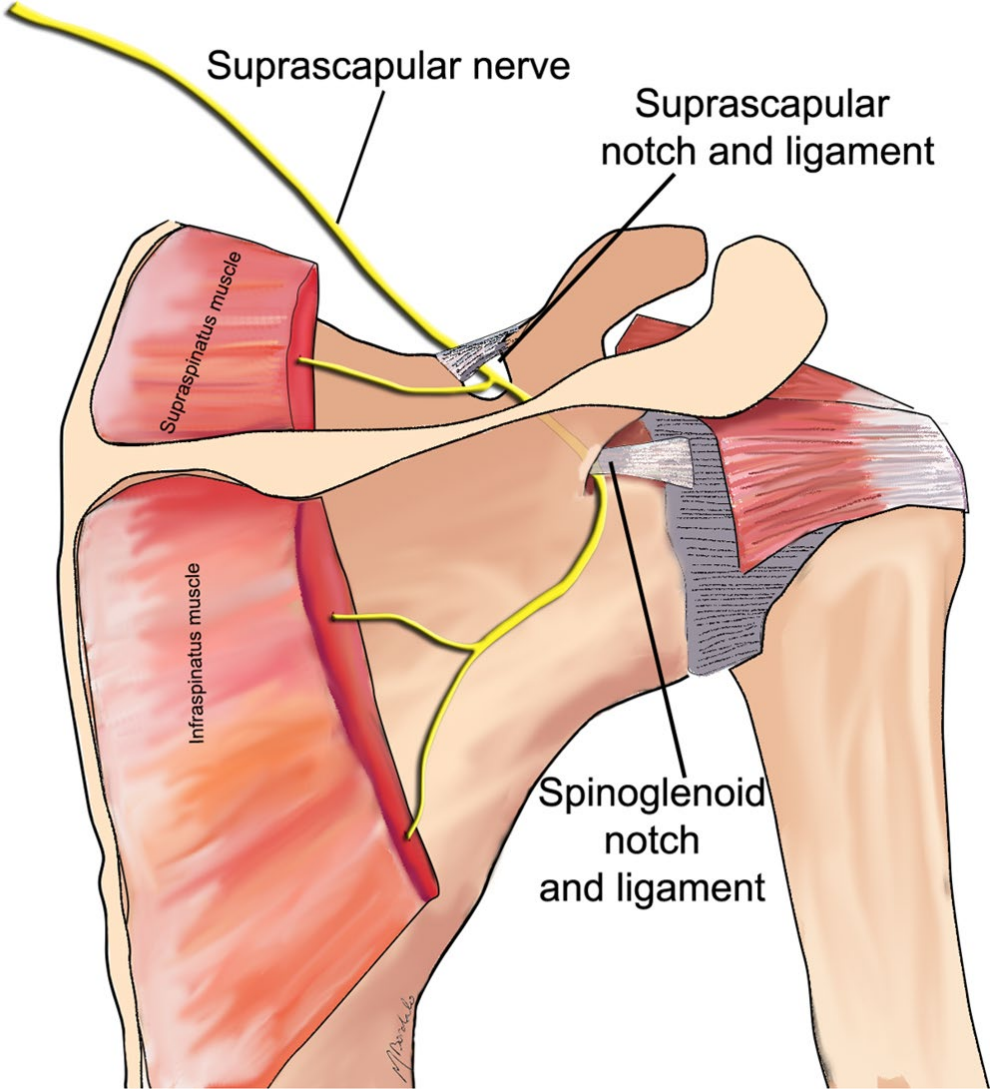
Schematic drawing of the suprascapular nerve anatomy and anatomic landmarks, especially with the suprascapular and spinoglenoid notches

The suprascapular nerve runs across the superior border of the scapula into the suprascapular notch, under the scapular superior transverse ligament (or suprascapular ligament). The nerve then runs posteriorly, supplies motor branches to the supraspinatus muscle, and enters the spinoglenoid notch under the scapular inferior transverse ligament (or spinoglenoid ligament), supplying motor branches to the infraspinatus muscle (Fig. 7).

**Fig. 8:**
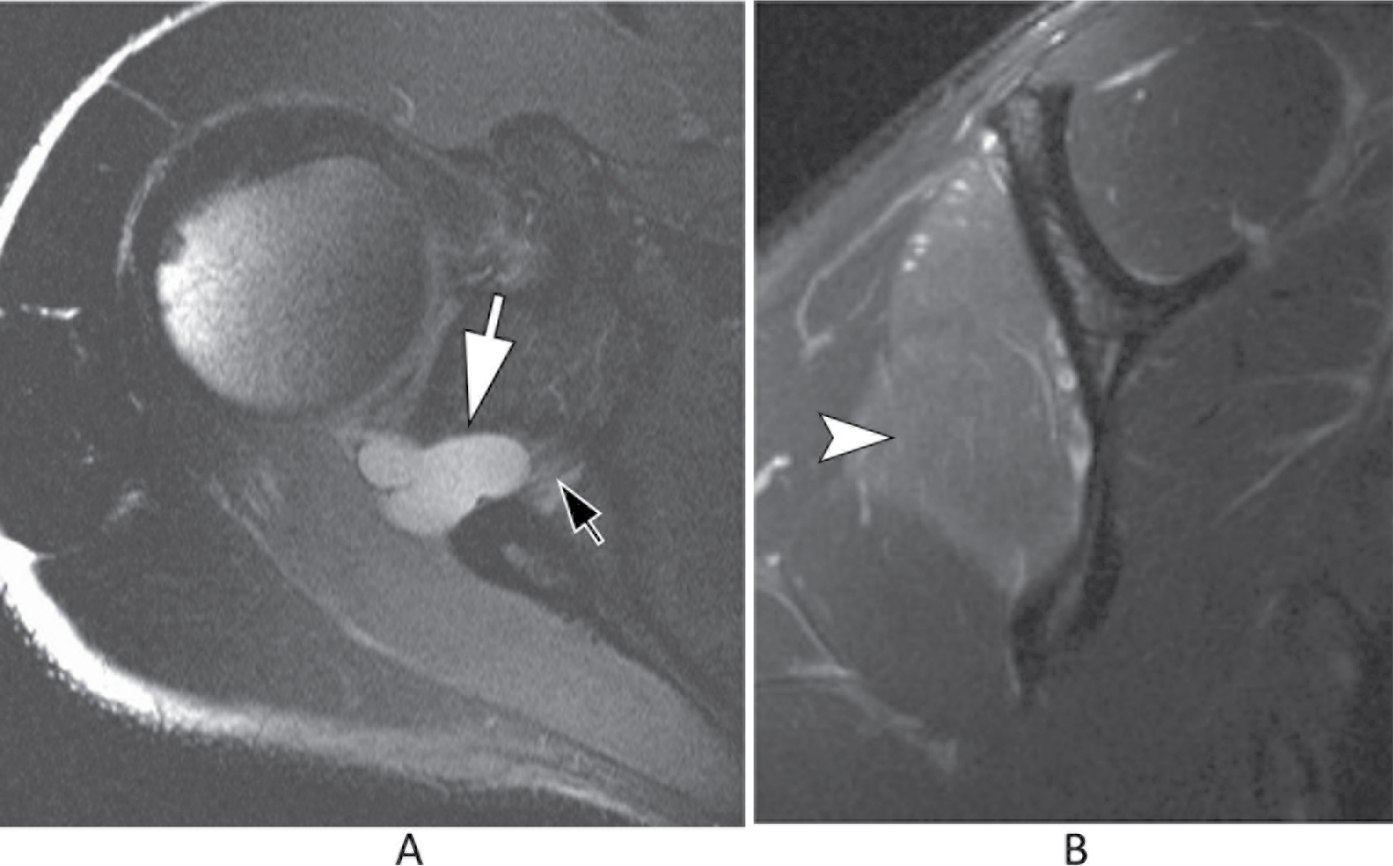
Suprascapular nerve compression. (**A**) Axial T2-weighted image of the shoulder. There is a paralabral cyst at the spinoglenoid notch (white arrow) with compression of the suprascapular nerve (black arrow). (**B**) Sagittal T2-weighted image with fat suppression shows denervation with edema at the infraspinatus muscle (arrowhead)

MRI is useful in evaluating the course of the nerve and patterns of muscle denervation. Entrapment of the nerve in the suprascapular notch, beneath the supraspinatus muscle, may lead to oedema and/or atrophy of both the supraspinatus and infraspinatus muscles. More distal entrapment in the spinoglenoid notch will result in selective denervation of the infraspinatus (Fig. 8) [24]. Visualization of the superior transverse ligament and its relationship to the nerve is also possible.

### Axillary nerve

Quadrilateral space syndrome (QSS) is a rare condition caused by compression of the axillary nerve and the posterior circumflex artery in the quadrilateral space. The clinical presentation of this condition can often be ambiguous, making magnetic resonance imaging (MRI) a valuable diagnostic tool.

**Fig. 9:**
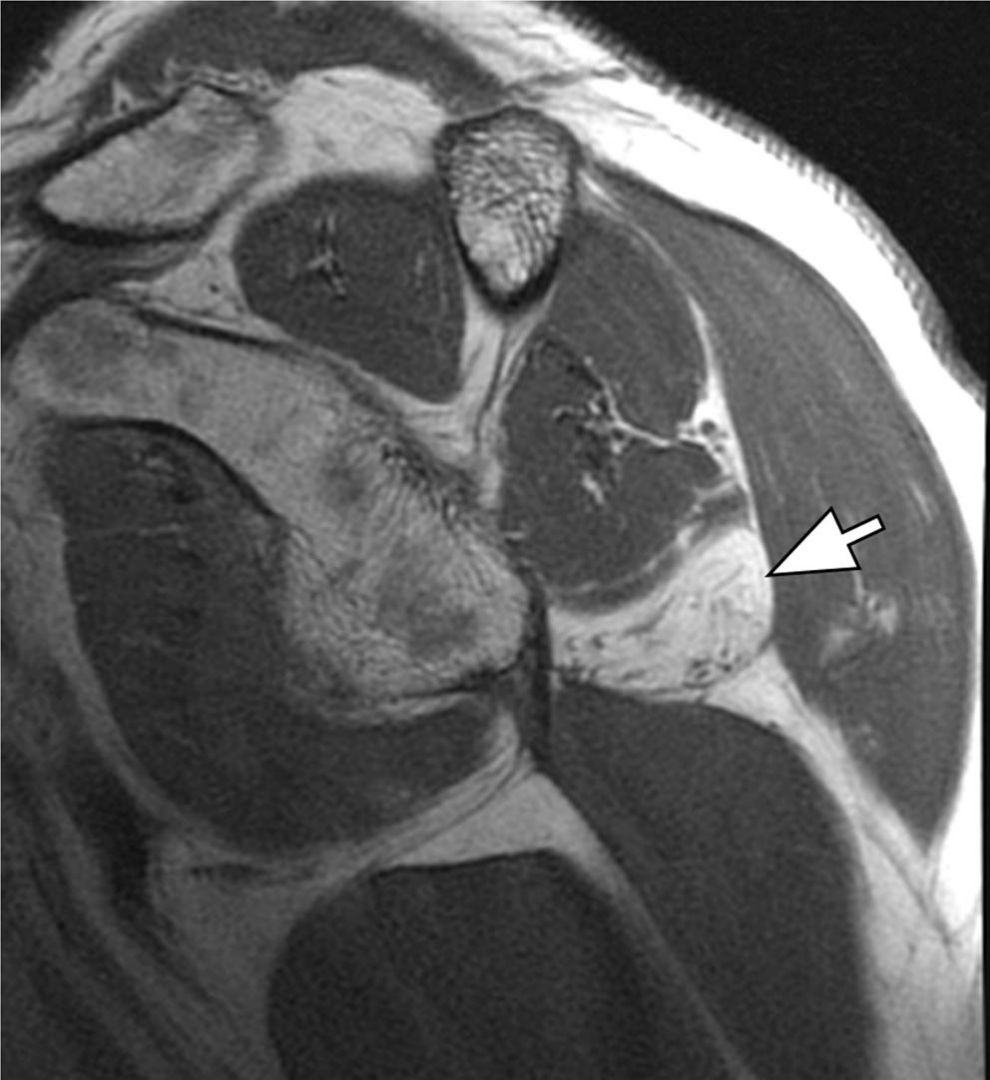
Quadrilateral space syndrome. Sagittal T1-weighted image of the shoulder. Complete fatty atrophy of the teres minor muscle (arrow)

**Fig. 10:**
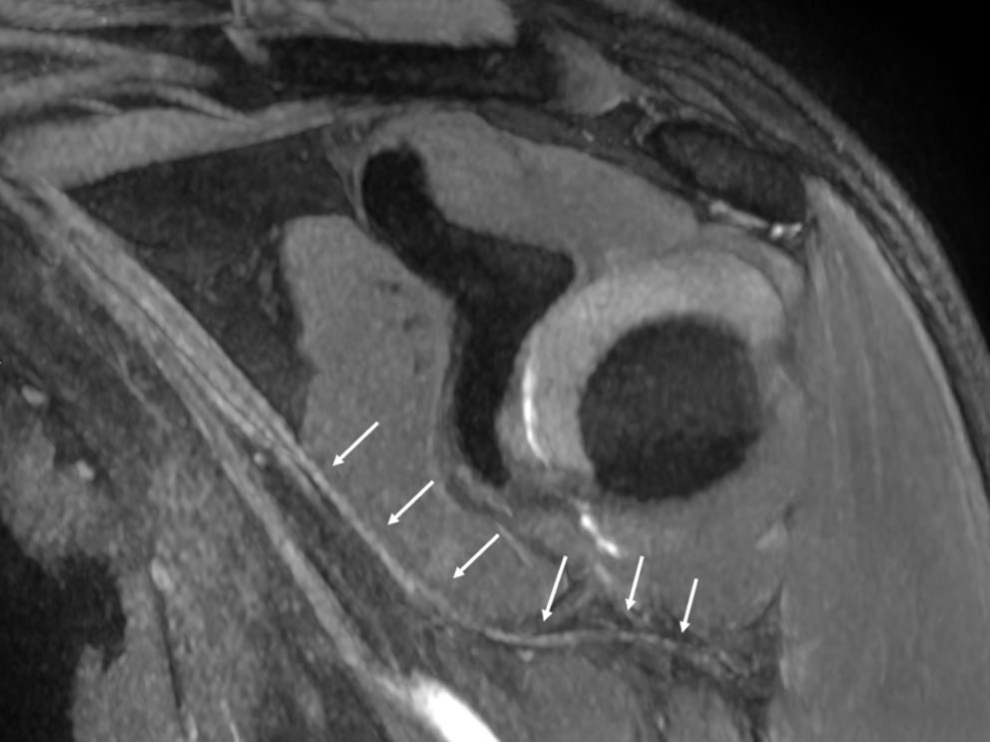
Quadrilateral space syndrome. MR neurography of the shoulder shows the axillary nerve (arrows)

Compression within the quadrilateral space may occur due to static or dynamic factors. MRI findings indicative of quadrilateral space syndrome (QSS) include increased signal intensity in the teres minor and deltoid muscles on T2-weighted images and/or evidence of fatty atrophy on T1-weighted images (Fig. 9). Isolated teres minor atrophy, which may occur in approximately 3% of routine MRI studies, can also be associated with other underlying conditions [25]. Additionally, MR angiography proves useful in identifying QSS by demonstrating occlusion of the posterior circumflex humeral artery during arm abduction and external rotation [26]. Advanced MR neurography techniques now allow for direct visualization of the axillary nerve, further aiding in the diagnosis (Fig. 10).

### Median nerve

**Fig. 11:**
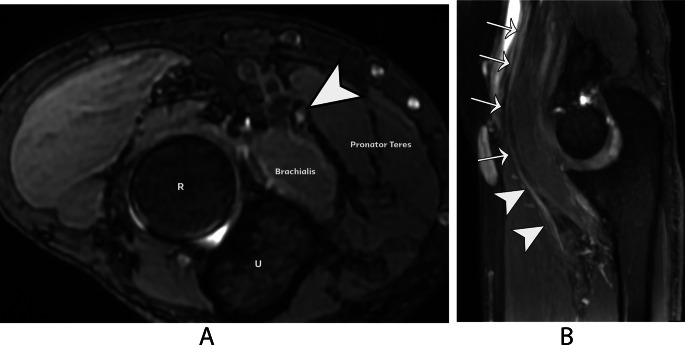
Lacertus syndrome. (**A**) Axial volumetric MR neurography sequence (steady-state) demonstrates thickening and an increased signal intensity of the median nerve (arrowhead) at the level of the pronator teres and brachialis muscles. R = radius, U = ulna. (**B**) Maximum Intensity Projection (MIP) sagittal reformatted image of the elbow reveals the median nerve. Its proximal segment (arrows) appears normal, while at the level of the pronator teres, the nerve exhibits thickening and an increased signal intensity, findings consistent with neuropathy (arrowheads)

Lacertus syndrome results from compression of the median nerve at the elbow, typically occurring beneath the lacertus fibrosus, between the superficial (humeral) and deep (ulnar) heads of the pronator teres muscle, or at the origin of the flexor digitorum superficialis muscle [27]. In our experience, MRI findings associated with lacertus syndrome may involve signal alterations within the median nerve at the level of the lacertus fibrous or flexor / pronator group with the use of high-resolution sequences (Fig. 11).

**Fig. 12:**
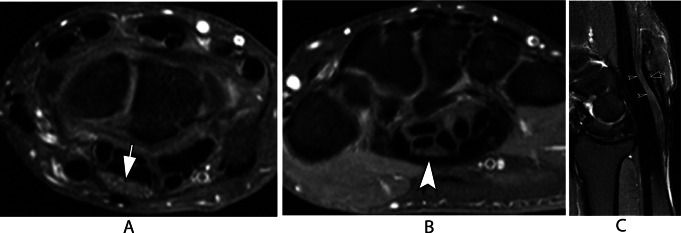
Carpal tunnel syndrome. Axial T2-weighted fat-suppressed images of the wrist at the levels of the pisiform (**A**) and the distal aspect of the carpal tunnel (**B**). Sagittal T2-weighted fat-suppressed image of the wrist shows the longitudinal axis of the median nerve (**C**). There is proximal enlargement (white arrow) and distal flattening (white arrowhead) of the median nerve. Note the increased signal and decrease in size with an “hourglass” appearance of the median nerve at the middle and distal aspects of the carpal tunnel (black arrowheads). There is also thickening of the flexor retinaculum (black arrow)

**Fig. 13:**
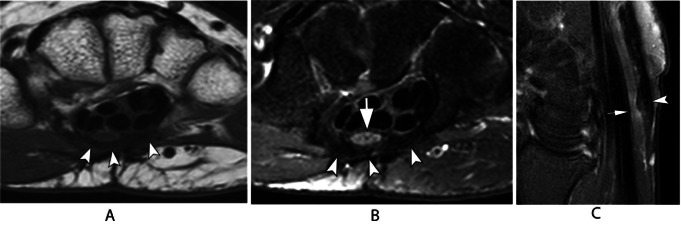
Post-operative carpal tunnel syndrome. Axial (**A**) T1-weighted and (**B**) T2-weighted fat-suppressed images of the wrist at the level of the distal carpal tunnel. Sagittal T2-weighted fat-suppressed image of the wrist shows the longitudinal axis of the median nerve. There is an incomplete flexor retinaculum release with fibrous tissue and thickening (arrowheads). The median nerve is flattened with increased signal (arrows)

Carpal tunnel syndrome (CTS) is the most common compressive neuropathy of the upper extremity, with a prevalence of 3% in the general population [28]. MRI has been shown to be accurate in 90% of CTS cases and to predict surgical benefit for these patients [29]. The most specific MR signs of CTS are proximal median nerve enlargement, flattening of the median nerve in the carpal tunnel, volar bowing of the flexor retinaculum, and T2 hypersignal in distal median nerve branches (Fig. 12). Isolated T2 hypersignal of the median nerve within the carpal tunnel has been reported to have a lower specificity [30]. Recently, diffusion tensor imaging has been reported in various studies as a potential tool in the diagnosis and evaluation of CTS treated conservatively [31]. Quantitative analysis is possible in CTS, and fractional anisotropy values of the nerve below 0.5 are considered abnormal [32]. MR neurography also plays an important role in the postsurgical evaluation of CTS by detecting incomplete flexor retinaculum release and signal alterations within the nerve (Fig. 13).

There are several quantitative criteria that can be measured in imaging exams and that have been described in the literature to aid in the diagnosis of CTS, but the most commonly used criterion is the sectional area of ​​the median nerve, particularly in the US literature.

Most authors suggest that the upper limit of normality of the median nerve is between 9 and 12 mm [2]. The value of 15 mm [2] has been suggested by some authors as the upper limit of normality for the sectional area of ​​the median nerve, serving to differentiate mild from severe cases of the disease [33].

### Ulnar nerve

**Fig. 14:**
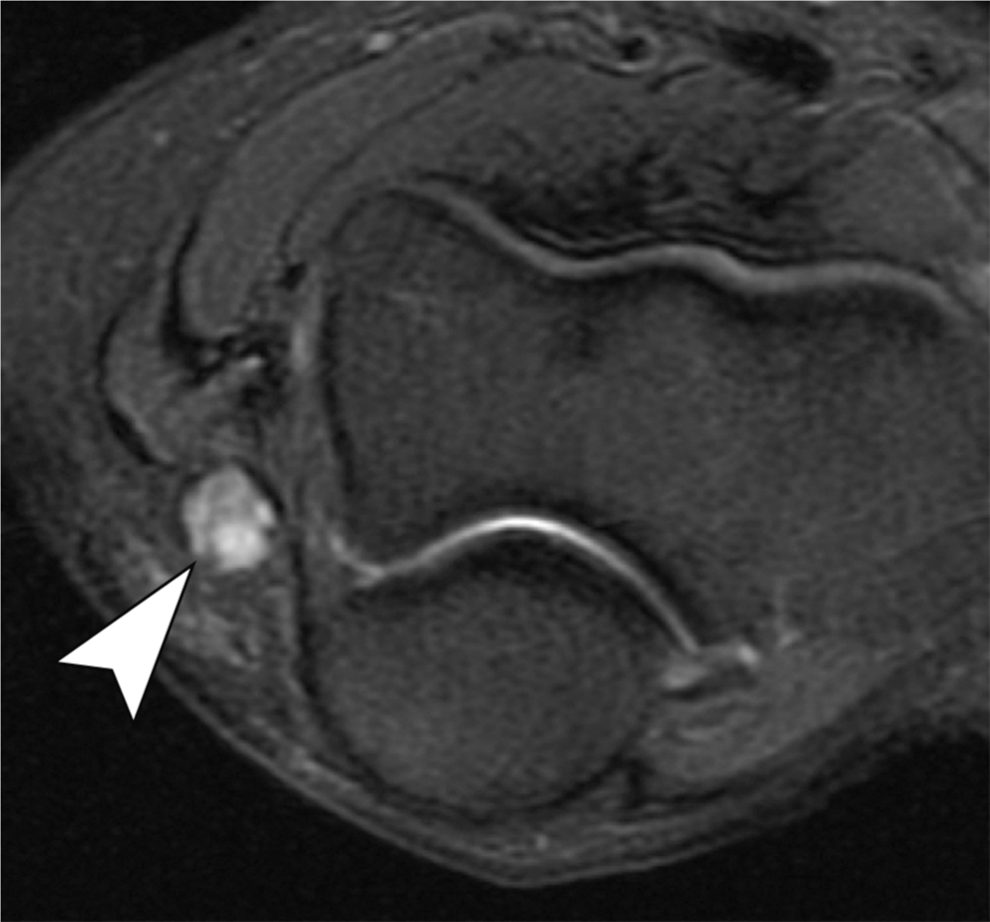
Ulnar neuropathy. Axial T2-weighted image with fat saturation. Thickening and increased signal of the ulnar nerve with loss of the regular fascicular pattern (arrowhead)

Compression of the ulnar nerve is the most frequently encountered neuropathy at the elbow and can result from both dynamic and anatomical factors. The cubital tunnel is the most common site of compression in this region, often caused by a reduction in tunnel volume during elbow flexion. MRI findings typically reveal increased signal intensity on T2-weighted images, nerve thickening (Fig. 14), and obliteration of fat tissue surrounding the nerve on T1-weighted images.

**Fig. 15:**
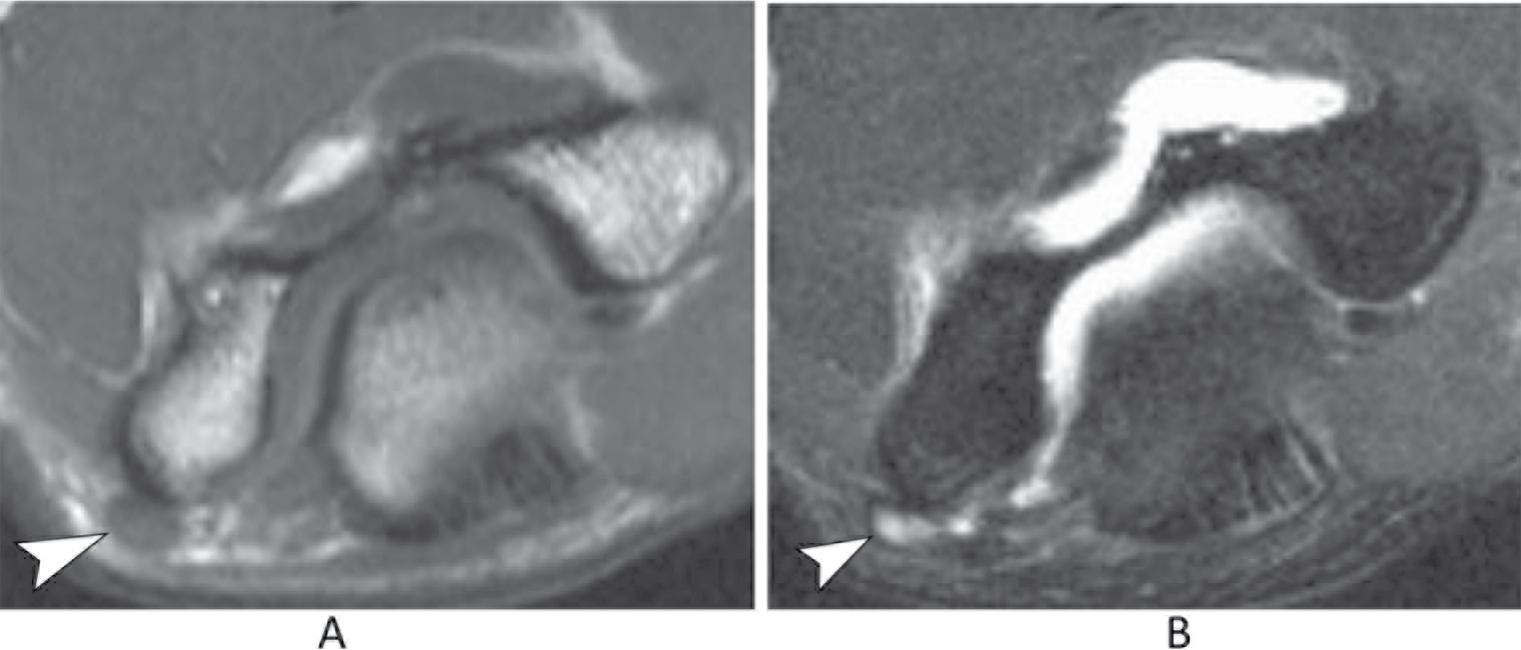
Ulnar nerve subluxation. Axial T1-weighted (**A**) and T2-weighted with fat suppression (**B**) images of the elbow demonstrate the ulnar nerve at the apex of the medial epicondyle (arrowheads), indicating subluxation. There is ulnar neuropathy associated, with increased signal and thickening of the nerve

**Fig. 16:**
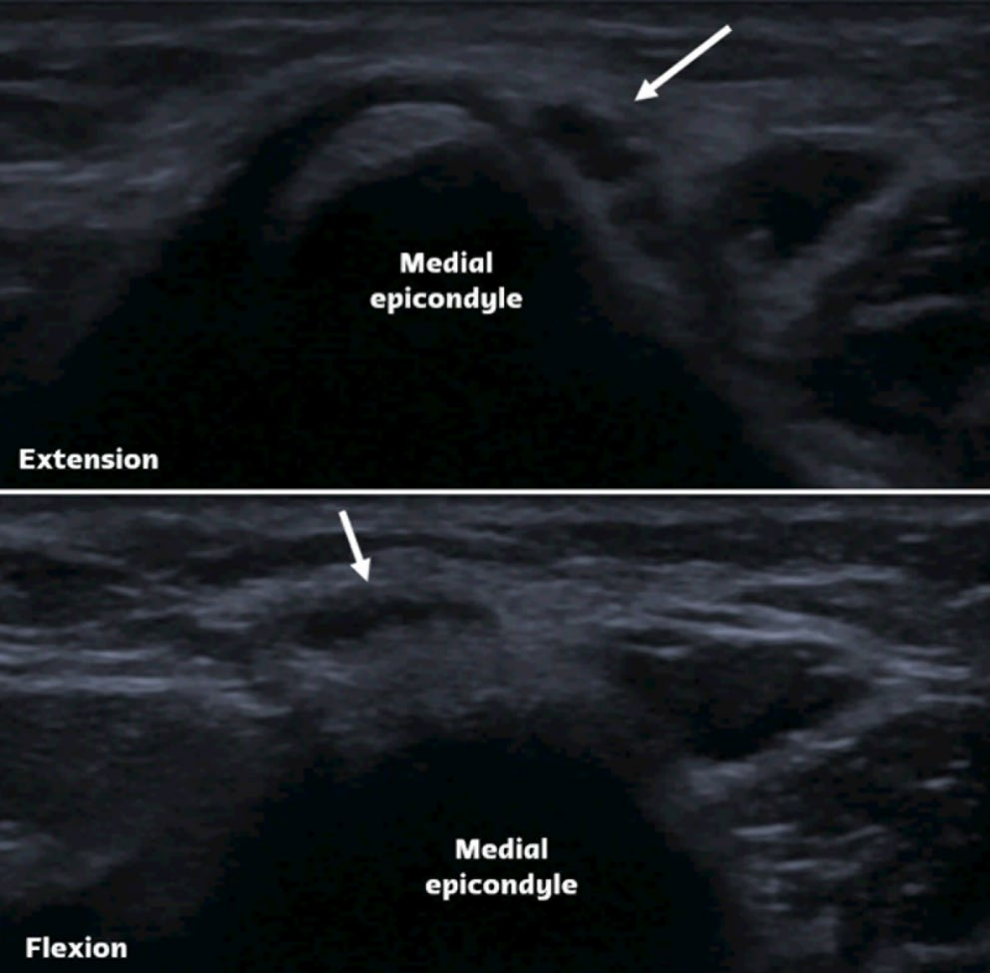
Ulnar nerve subluxation. Ultrasound images of the elbow in extension and flexion demonstrate the positional change of the ulnar nerve. In extension, the ulnar nerve is visualized posterior to the medial epicondyle (arrow). During flexion, anterior subluxation of the ulnar nerve occurs, with the nerve translocating beyond the apex of the medial epicondyle (arrow)

Ulnar nerve subluxation is a relatively common finding, occurring in up to 16% of individuals without symptoms [34]. In some cases, it can lead to compressive neuropathy. While subluxation can be observed on standard MRI scans, imaging performed during elbow flexion provides improved visualization of nerve dislocation. (Fig. 15) [35]. Dynamic evaluation is more effective in detecting nerve instability, and ultrasonography (US) is an appropriate imaging modality for assessing this condition and associated snapping syndromes (Fig. 16) [36]. Dynamic maneuvers, including flexion, extension as well as flexion against resistance, are recommended to improve the likelihood of achieving an accurate diagnosis.

**Fig. 17:**
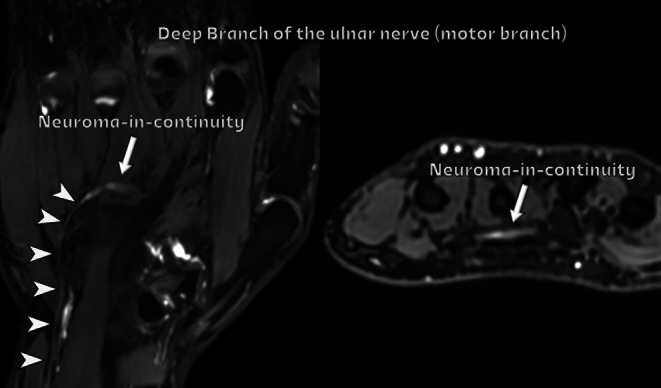
Ulnar nerve injury at the wrist. The patient presented with a prior carpometacarpal dislocation, leading to subsequent motor dysfunction of the distal ulnar nerve. MR neurography images of the hand (steady-state sequence), reformatted in coronal and axial planes, demonstrate the normal appearance of the proximal ulnar nerve (arrowheads). In the distal deep motor branch, there is evidence of a complete nerve transection with the formation of a neuroma-in-continuity (arrows)

Ulnar nerve compression can occur at the wrist level. Within Guyon’s canal, the ulnar nerve divides into superficial sensory and deep motor branches. On MR, the normal anatomy is well depicted on T1-weighted images, and bifurcation of the superficial and deep nerve branches is adequately seen [37]. MR-neurography can identify abnormalities along any portion of the ulnar nerve, including its deep branch (Fig. 17). Ulnar nerve fascicular pattern and caliber alterations are better visualized on US.

### Radial nerve

**Fig. 18:**
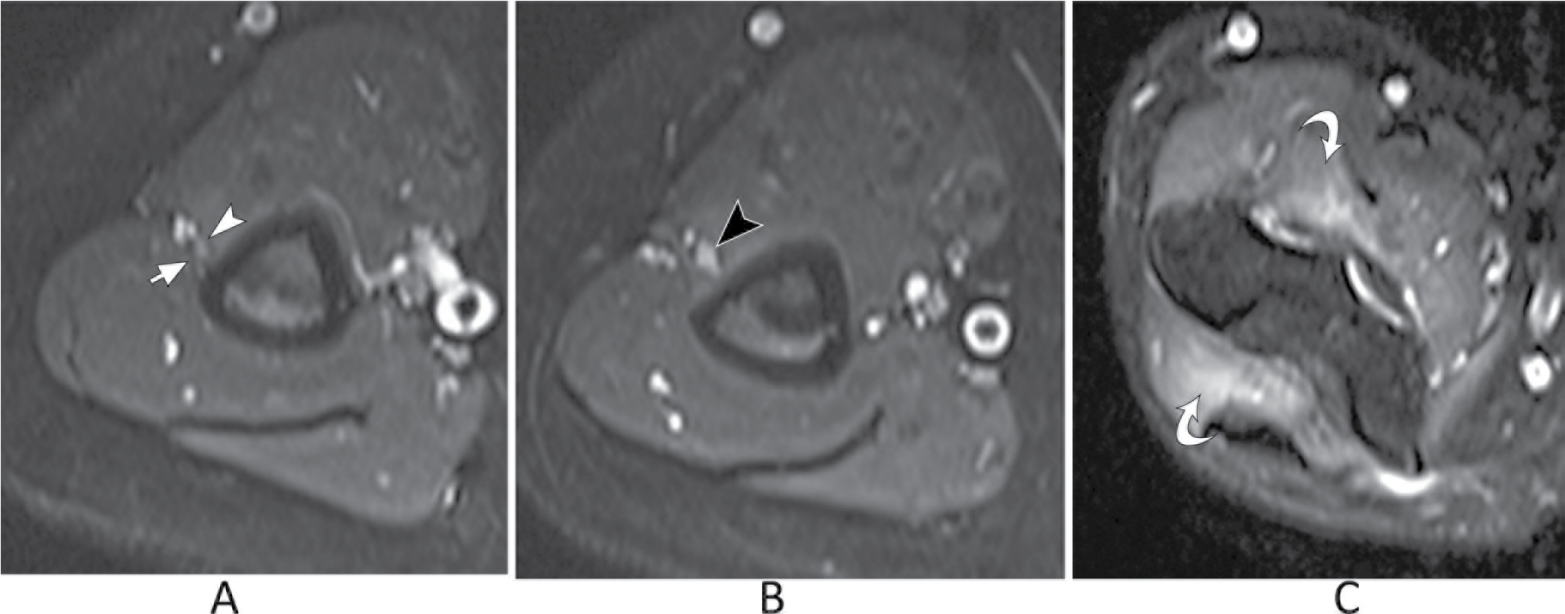
Radial nerve compression at the spiral groove. Axial T2-weighted fat suppressed images at the level of the humeral spiral groove (**A** and **B**) and the level of the elbow joint (**C**). In (**A**), the ulnar nerve has normal size and signal (white arrowhead) and is starting to cross the lateral intermuscular septum (white arrow). At a slightly more distal image, there is increased signal and thickening of the radial nerve (black arrowhead). There is denervation edema of the brachialis and supinator muscles (curved arrows)

**Fig. 19:**
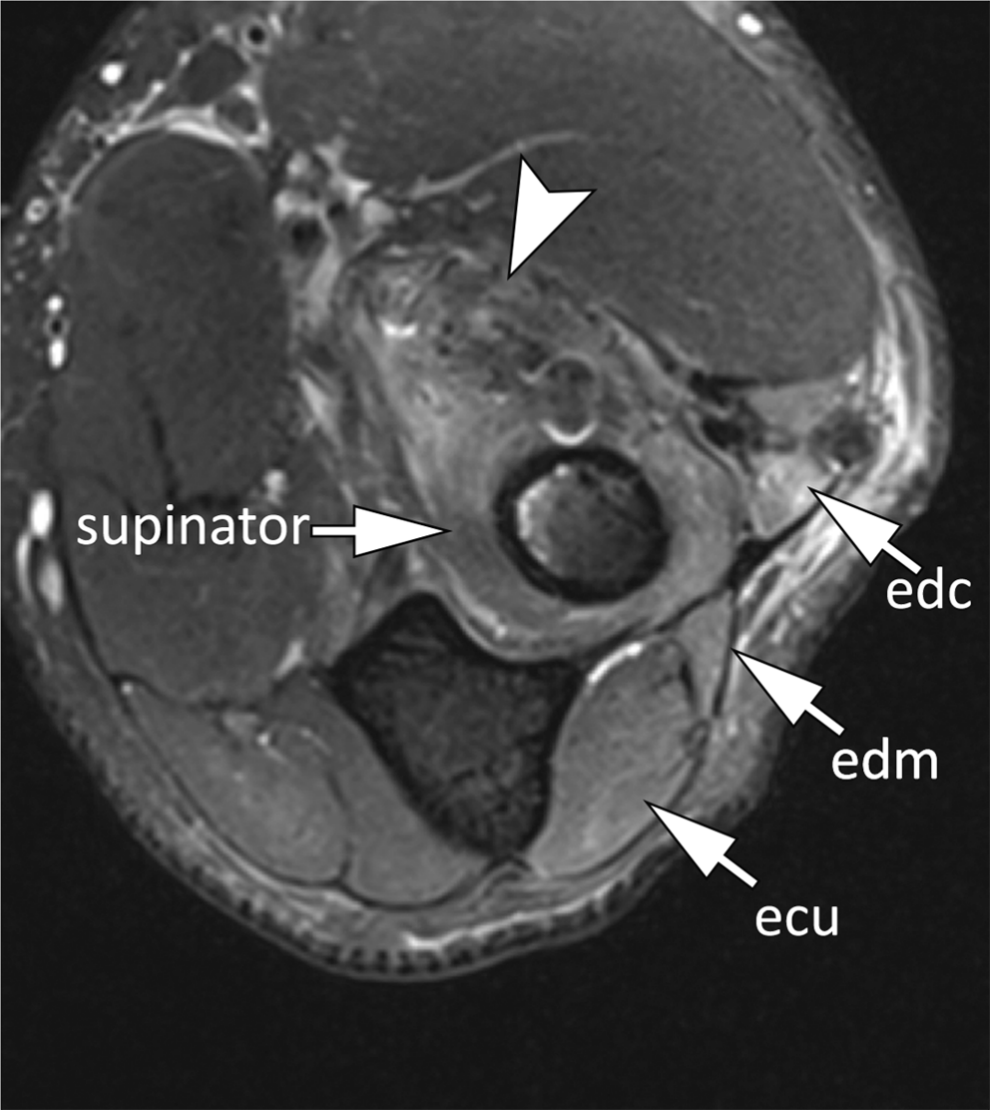
Posterior interosseous neve entrapment. Axial T2-weighted image with fat saturation demonstrates a postoperative scar tissue at the anterolateral aspect of the elbow (arrowhead) with obliteration of the posterior interosseous nerve. There is denervation muscle edema of the supinator, extensor carpi ulnaris (ecu), extensor digitorum minimi (edm) and extensor digitorum communis (edc) (arrows)

Radial nerve entrapment is the least frequent form of nerve compression and is often linked to trauma. In the arm, compression may occur at the humeral spiral groove as the nerve transitions through the lateral intermuscular septum into the anterior compartment. Utilizing 3D volumetric imaging, MR neurography can visualize radial nerve thickening at the lateral intermuscular septum, along with abnormalities in its caliber and signal (Fig. 18) [34, 38]. At the level of the elbow joint, the radial nerve bifurcates into the superficial radial nerve, a sensory branch, and the posterior interosseous nerve (PIN), a motor branch. The PIN passes under the arcade of Frohse, a fibrous arch present in 35% of individuals and formed by the proximal thickened edge of the superficial head of the supinator muscle, which is the most common site of compression of the PIN [39]. Compression of the PIN may lead to a denervation pattern in the muscles innervated by the PIN, namely, the supinator, extensor digitorum communis, extensor digitorum minimi, and extensor carpi ulnaris (Fig. 19).

## Summary

MR neurography and high-resolution US are excellent techniques for the evaluation of peripheral nerves. Knowledge of currently available techniques is essential to perform state-of-the-art MR and US examinations. To provide an accurate diagnosis, physicians must also be familiar with the basic clinical aspects of nerve entrapment and, especially, with anatomic and pathologic aspects related to the nerves.

## Data Availability

No datasets were generated or analysed during the current study.
